# Fungal food spoilage of supermarkets’ displayed fruits

**DOI:** 10.14202/vetworld.2019.1877-1883

**Published:** 2019-11-29

**Authors:** Iman Saleh, Roda Al-Thani

**Affiliations:** Department of Biological and Environmental Sciences, College of Arts and Sciences, Qatar University, P.O. Box 2713, Doha, Qatar

**Keywords:** country of origin, fungi, post-harvest, shelf-life, spoilage

## Abstract

**Background and Aim:**

Post-harvest fungal infection of fruits and vegetables is mainly caused by fungal pathogens that can be harmful to both human and animals as they produce mycotoxins, post-harvest diseases in fruits and vegetables are a serious problem that results in the loss of a large percentage of crops reaching 50% in some fruits. This study aims at screening the post-purchasing shelf-life of four highly consumed fruits and vegetables and at identifying the fungal strains behind their spoilage in Qatar.

**Materials and Methods:**

Fruits and vegetables were collected from the market to study their post-purchasing shelf-life and to identify the fungal types involved in samples rotting. Factors that affect samples’ shelf-life were also analyzed.

**Results:**

A total of 73 fungal isolates were isolated and identified, with the highest percentage of *Penicillium* (21.9%) followed by *Rhizopus* (17.8%). Interestingly, many mycotoxins producing and diseases inducing fungi were identified in this study; this includes *Rhizopus*, *Aspergillus*, *Penicillium*, *Alternaria*, *Fusarium*, *Cladosporium*, *Botrytis*, *Geotrichum*, and *Colletotrichum*. Statistical analysis shows that different fruits have significantly different shelf-life and different predispositions for spoilage. In many cases, a strong relationship was shown between the fungal types isolated and the country of origin of the fruit. Finally, the price of the commodity did not have a significant effect on its contamination level nor did the market from which the sample was purchased. This indicates that the fruit displaying methods in Qatar do not affect their contamination level.

**Conclusion:**

The study is among the first reports about fungal types involved in fruits and vegetables rotting in Qatar and it highlights the strong link between spoiling fungi and their country of origin.

## Introduction

Microbiological food safety is a major economic and public health concern nowadays. According to the WHO, one in every 10 people become ill from consuming contaminated food each year, a trend that results in the death of 420,000 individuals annually WHO [1]. Food contaminants consist of physical agents, including pieces of metals or plastic, that enter food during the packaging stage of manufacture, as well as other chemical agents including heavy metals, pesticides, and, most importantly, microbes [2,3].

Fruits are a major source of nutrients for humans and animals, but it has been reported that, globally, around 45% of harvested fruits and vegetables are wasted every year due to spoilage caused by contaminated growth environments, inappropriate harvesting conditions, unsafe handling and storage processes, and incorrect methods of display [3]. Even though freshly harvested vegetables and fruits are unavoidably contaminated with a variety of bacteria, fungi and other microorganisms. However, molds in general and mycotoxin producers in particular are the main cause of spoilage, especially in products that are refrigerated in open boxes [4-6].

Microorganisms, including bacteria and fungi, cause considerable economic losses by spoiling not only harvested fruits and vegetables but also crops in their fields. The identification of such spoilage microorganisms is a crucial step toward controlling them. Some pathogenic strains specific to fruits are pathogenic to humans as well, especially those that produce toxins [5,7]. The metabolites of many such microorganisms are heat stable, which suggests that they remain in the food after heat processing and continue to cause toxicity. Once mycotoxins are formed, it is difficult to manage their quantities as they are stable under storage conditions and particularly insensitive to physical and chemical treatments. Therefore, the best way to limit mycotoxin exposure is to stop them from forming in the first place [6,8,9].

Different spoilage-causing microorganisms have different nutrients requirements. Due to the variable composition of fruits and vegetables, it is important to determine the microbial hazards for each product separately. In Qatar, the country imports over 90% of its consumed food. Food security as one of the major challenges of the modern world is mentioned as one of the pillars of Qatar’s National Vision 2030, which places great importance on fungal contamination control. Products of great concern include cucumbers and tomatoes, which are produced in large amounts in Qatar and are among the most highly consumed vegetables in the Middle East, as well as oranges and strawberries, which are used in great quantities for the production of fresh juices. Therefore, it is of significant concern to understand the spoiling agents behind their shelf-life termination [10,11].

In this study, samples of cucumber, tomato, strawberry, and orange were tested to determine their shelf life and to identify spoiling agents, if found. Factors that affect the rate of fungal spoilage in the purchased samples were also analyzed.

## Materials and Methods

### Ethical approval

This study did not involve the use of live animals, and hence, ethical approval was not required.

### Sample collection

Samples were collected during four trips which took place between September 2017 and November 2017. All trips occurred on Saturday and samples were collected between 10.00 am and 12.00 pm from three large supermarkets in Qatar. At each market, a sample was collected per available type of cucumber, tomato, orange, and strawberry. Samples were handled aseptically, kept in sterile bags with a breathable patch 44 cm×20.5 cm (Sun bag, transparent, SIGMA-ALDRICH, Montreal, QC), and were assigned serial numbers. All necessary data regarding each sample were collected including the product’s country of origin, its type, and the price per kg. Samples were kept in the sterile bags and at a storage temperature approximating that of the supermarket until transferred to the laboratory the next day.

### Sample processing

The sample collection was kept individually in a sterile and empty Petri dish with a sample number identifier. All samples were incubated at 25°C and observed twice a day to determine when each would rot. When a sample began to exhibit fungal hyphae growth, it was removed from the incubator, at which point, the observer noted the day as the end of the home shelf-life of that particular sample. Hyphae were subsequently collected using a sterilized needle and subcultures on potato dextrose agar (PDA) [12]. The number of spoiling spots on each sample was counted as a measure of contamination level. If more than spoiling spot color and shape appear, each spot’s hyphae were cultured separately. Rotten fruits were discarded in hazardous waste and the inoculated PDA plates were separately incubated at 25°C. The process continued for 10 days, after which time, the experiment was completed.

### Identification of isolated fungi

After 1 week of incubation, fungal isolates were identified using colonies and cell morphological features such as the thallus growth pattern, pigmentation, conidiophore, and conidial morphology [13]. Isolated fungi were identified using cotton blue in lactophenol stain. A drop of the stain was placed on a clean slide and a portion of the mycelia was placed on the stain using a sterilized needle and forceps. A coverslip was then placed on the wet mount and the slide was examined by a light microscope at various magnifications. The morphology and characteristics of the conidia and conidiophores were then used to classify the different types of fungus according to the standard taxonomic system [5]. Data were entered into the excel datasheet, pictures were taken of the fungi on the PDA agar, and pictures of each isolate under the microscope showing conidia and conidiophores were taken.

### Statistical analysis

Data were analyzed using SPSS statistical software, version 24 (IBM Corp., NY, USA). Chi-square test and ANOVA were applied against an acceptance limit of p<0.05. Chi-square was used to determine if there were significant differences in the detection of spoiling among the four samples type, the three collection markets, the eight countries of origin, and the two levels of the countries’ economic conditions and commodity price.

## Results

### Fungal isolates

Out of the 90 samples, 51 (56.7%) showed fungal growth within the experiment timeline (10 days). Various types of fungus were isolated from different fruits. Table-1 shows the number of various fungal types isolated from each kind of fruit. A total of 73 fungal isolates were collected and identified, with the highest percentage of *Penicillium* (21.9%) followed by *Rhizopus* (17.8%). Different mycotoxins producing fungi were isolated in the study.

**Table-1:** The number of various fungal types isolated from each kind of fruit.

Fruit type	*Aspergillus*	*Rhizopus*	*Penicillium*	*Cladosporium*	*Botrytis*	*Alternaria*	*Fusarium*	Others*	Number of collected samples
Cucumber	1	2	4	1	0	4	5	1	16
Orange	2	0	4	0	0	1	0	1	30
Strawberry	5	8	3	1	5	0	0	0	14
Tomato	2	3	5	4	2	5	0	4	30
Total	10	13	16	6	7	10	5	6	90

* Mucor was isolated from cucumber sample, *Colletotrichum* was isolated from orange sample, *Geotrichum candidum* was collected from a tomato sample

### Samples contamination rates

Chi-square test was used to detect the factors that significantly affect fruit spoilage: Sample type, the market from which the samples were purchased, and the sample’s country of origin. Our results show that 100% of the collected strawberry samples became rotten within the 10 day experimental timeframe followed by 81.25% of the cucumber samples. On the other hand, 60% of the tomato samples became rotten during the experiment, while only 20% of the oranges showed fungal growth within the same time period. The results of the Chi-square test indicate that the fungal-driven spoiling rates of the various fruits are significantly different (p=0<0.01).

### Effect of displaying market on fruit spoilage

Samples were collected from three large supermarkets in Doha. A Chi-square test demonstrated no significant difference among the different markets in terms of the number of samples showing fungal spoilage within 10 days (p=0.87>0.05), which implies that contamination is country of origin related and not induced in market.

### Effect of fruits country of origin on fruit spoilage

When testing for the effect of the country of origin on commodities’ fungal spoilage, the Chi-square p-value obtained was 0.02 (p<0.05); however, the Chi-square assumptions in this case were not valid (10 cells have expected count <5%). Chi-square test results of the comparison of the number of samples showing fungal growth among samples originated from different countries suggest that the country of origin has an effect on the contamination of commodities, although significance could not be proven, as more samples were needed to reach an adequate level of statistical power. The effect of the country of origin can be observed when analyzing the results; for example, samples imported from the Netherlands were highly contaminated with *Penicillium* when compared to samples from other countries. On the other hand, *Penicillium* is known to be more common in citrus fruits. However, the data in Table-1 do not demonstrate a significant difference between the number of *Penicillium* isolated from oranges and those isolated from the rest of the fruits, which might link *Penicillium* to the country of origin.

The data indicate also high concentrations of *Cladosporium* in samples originating specifically from Morocco. Interestingly, a total of 16 samples of cucumber were collected from Iran (six samples), Qatar (six samples), and Lebanon (four samples). Out of the six locale samples, five were contaminated with *Fusarium*, suggesting that most *Fusarium* isolates are local in origin. Finally, it is worth mentioning that the rate of occurrence of *Aspergillus* and *Rhizopus* is particularly high in samples originated from the USA. However, this high rate might be more related to the fruits’ nature than to the country of origin, as those two fungal types were linked to strawberry infections knowing that most of these fruit samples were of American origin (data not shown).

### Effect of the country of origin’s economy on fruit spoilage

The various countries of origin of the samples were divided into countries categorized as having a developing economy and those with a developed economy (as based on the Department of Economic and Social Affairs of the United Nations Secretariat). Chi-square test was again applied to determine whether a countries’ economic classification affected the fungal contamination frequency of their fruits. Although the test returned a non-significant p=0.081 and >0.05, Table-2 indicates that 49% of the samples originating from developing economy countries were contaminated, while 67.6% of samples from developed economy countries showed fungal growth within 10 days. The statistical results are likely to be biased by the fact that different fruits were exported from specific countries; for example, strawberries are more prone to spoilage than other fruits and, due to the nature of their skin, tend to be produced in developed nations.

**Table-2:** Number of samples originated from countries of different economy and their contamination frequency.

Contamination	Economy		Total

	Developed	Developing	
No	12	27	39
Yes	25	26	51
Total	37	53	90

### Effect of price of commodity on spoilage rate

The collected fruit samples ranged in price from items that were on promotion, to very expensive fruit types. Therefore, it is important to interrogate the effect of the price of fruit on their home shelf-life to understand if, in the market, low price reflects the microbiological condition of the commodity or if it is a reflection of the taste and/or probable variation in the nutritional value. The average price per commodity was calculated by SPSS, as shown in Table-3.

**Table-3:** Descriptive statistics of the prices QAR/kg of different fruit types.

Sample type	Mean	n	Standard deviation
Cucumber	8.20	16	4.38
Orange	8.85	30	5.46
Strawberry	63.02	14	18.30
Tomato	16.31	30	6.81
Total	19.65	90	20.96

High and low price categories were created by considering fruits whose price was above or below the average for their category respectively. Chi-square test was computed to assess the effect of price category on spoilage rate. Chi-square results are for cucumber (p=0.267>0.05), orange (p=0.232>0.05), and tomato (p=0.296>0.05) indicate a non-significant effect. The test could not be conducted on strawberries because all samples were contaminated. In the three categories of fruits tested, the data did not provide evidence that more expensive fruits are less contaminated or last longer. Instead, price variation may be a product of the quality of the commodity, rather than its home shelf-life.

### Number of fungal types per sample

**Figure-1:**
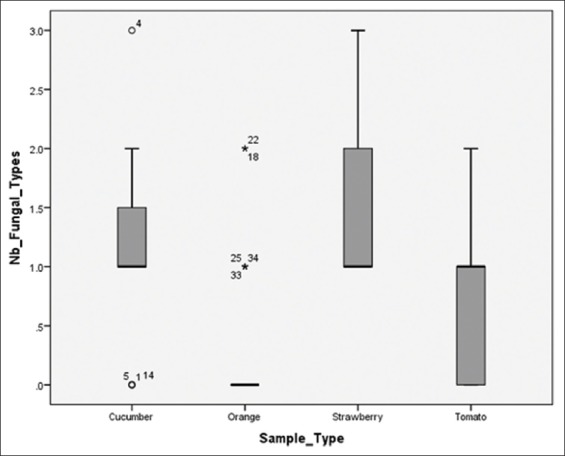
Box plot of the number of fungal contaminants by sample type.

Some fruits were found to be contaminated with more than one, but no more than three, types of fungi at once. Data were summarized in a boxplot and the results indicate that strawberry samples contain the greatest variety of fungi of all the fruits. This range of contamination is due to the nature of the skin of this fruit, which contains pouches that fungal spores get stuck within. It should be noted that strawberries were primarily contaminated with black molds, whose spores can easily be transmitted by air. The cucumbers data demonstrate the existence of some outliers, but its median is at one fungal type per sample, whereas tomatoes showed high specificity in terms of fungal types and oranges did not have enough contaminated samples to be included (Figure-1).

The P-P plot of the number of fungal types indicates that the number of fungal types per fruit sample follows a normal distribution (data not shown). SPSS was, therefore, used to perform an ANOVA test to compare the mean numbers of contaminant kinds per sample type. The results (p<0.01) suggest that fruits do significantly vary in the number of fungal types they carry. *Post hoc* tests show that the main significant differences in the number of fungi are between cucumbers and oranges, oranges and strawberries, tomatoes and oranges, and tomatoes and strawberries.

### Contamination level of various fruit samples

The level of contamination across different fruits was assessed based on the number of spots shown on each sample at the beginning of the rotting process. Because each spot represents a spore, their number reflects the level of contamination. The levels were described as follows:

1. Undetectable contamination level: Zero spots (within 10 days)
2. Low contamination level: 1-4 spots
3. Moderate contamination level: 5-10 spots
4. Heavy contamination level: Sample fully covered with fungus
5. Detected by swab: Contamination was not visible, but proven by swab culture.

**Figure-2:**
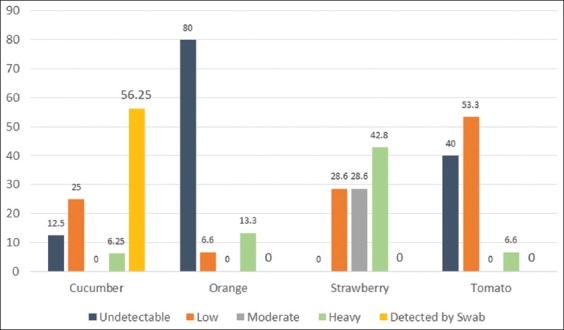
The percentage of various fruits that have undetected, low, moderate, heavy, and swab detected contamination levels.

Figure-2 summarizes the contamination levels of each of the fruits. Strawberries exhibited the highest levels of fungi per fruit; again, this may result from the characteristics of its skin. Oranges exhibited a high level of contamination across 13.3% of samples, whereas more than half of the cucumber samples (56.25%) showed no hyphae growth by the end of the 10 days at all. However, the visibly bad condition of the fruit motivated us to take a swab culture from them on PDA, all of which demonstrated the growth of various fungi types. Finally, it is worth mentioning that 60% of the tomato samples were contaminated but that most of those (53.3%) exhibited only a low spread of contaminations per fruit, which can be explained by the smooth, thick nature of the tomatoes’ skin.

### The home shelf-life of various fruits

The time taken by a fruit sample to rot will vary from 1 to more than 10 days. Based on the rotting speed, samples were divided as follows:

1. Speed 1 samples: Very short home shelf-life, rotten between 1 and 3 days
2. Speed 2 samples: Short home shelf-life, rotten between 4 and 6 days
3. Speed 3 samples: Moderate home shelf-life, rotten between 7 and 10 days
4. Speed 4 samples: Long home shelf-life, the samples did not rot within the experiment time.

**Figure-3:**
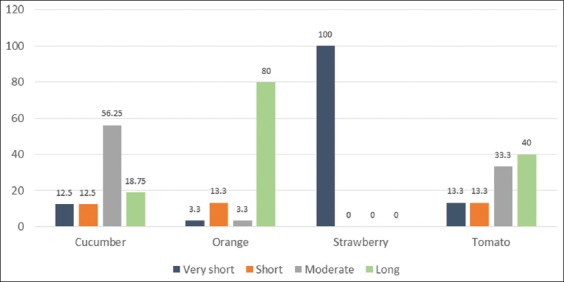
Percentages of various fruits that have very short, short, moderate, and long home shelf-life.

Strawberries experienced the shortest home shelf-life, with all samples rotting within 3 days. This period was followed shortly by cucumbers, of which 87.5% of the samples were in a bad condition within 10 days. About 40% of the tomato samples and 80% of the orange samples remained in a good and appealing condition during the 10 days of the experiment (Figure-3).

## Discussion

Fungi have always been a public health concern. In 1992, a study conducted in Australia on packaged cheddar cheese showed that mold infections were tough to control as they were isolated from different equipment in the cheese factory, fungi were also isolated from the air, curd, and whey. The most common fungi isolated from the packaged cheese samples were *Cladosporium* followed by *Penicillium*, which are our most commonly isolated fungi [14]. Similarly, in 2001, Weidenborner determined the major source of contamination for pine nuts as *Cladosporium*. A total of 31 different species were isolated in the study, of which 16 were potentially toxigenic [15].

Recent studies regarding fungal contamination are conducted all over the world. A total of 117 isolates of fungi were recovered from nuts and dried fruits in the Washington D.C. area, including potential toxigenic *Aspergillus*, *Penicillium*, *Alternaria*, and *Fusarium* species. Similarly, the most common isolated fungi type identified in a study by Tournas *et al*. [16] was *Aspergillus*, while the most common mold in walnuts was identified as *Penicillium*. In Brazil, a 2007 study investigating toxigenic fungi on dried fruits identified *Aspergillus niger* as the most common species from a sample of 117 [17]. Both studies imply an affinity between *Aspergillus* and dried fruits.

Similarly, a study of grapes conducted in Lebanon identified a high level of contamination with various strains of *Aspergillus* section Nigri. In addition, tests of ochratoxin A (OTA) levels indicated low-level contamination of the isolates in 57.4% of cases which, again, illustrates the risk to human health posed by the toxins produced by *Aspergillus* species, the third most isolated species in the present study [13].

Aflatoxin and OTA producing fungi are also of major concern in cocoa production. During the chocolate production process but, in particular, during the fermentation of the cocoa beans, molds contamination is common [18]. In a study conducted in Brazil, in 2014, out of 356 chocolate samples tested, 303 (85.1%) showed traces of OTA. Although the detected toxin levels were within acceptable ranges, chocolate-based products are widely consumed, especially by children, and the accumulative effect of low-level OTA consumption may cause severe health problems in vulnerable populations, making the control of toxin-producing fungi of major importance [19].

While the orange samples considered in the present study displayed the highest level of contamination with *Penicillium*, the previous study conducted on sweet oranges in Nigeria found isolates of *A. niger* to be the most numerous (27.5%) followed by *Rhizopus* (22.5%) and *Aspergillus flavus* (17.5%) [20]. In close consistency with our results, the second and third most common spoiling agents of our study were isolated in Uyo metropolis, Nigeria, from samples of cucumbers, carrots, cabbages, and onions. Approximately one-third or 37.5% of the Nigerian samples showed contamination with *Rhizopus stolonifer* and with *Aspergillus fumigatus*, while *A. niger* was found only in cucumber and onion samples, which constituted 25% of the overall number of samples [21]. Eight of our 11 *Botrytis* isolates were isolated from the strawberry sample, which is consistent with a study conducted in Virginia USA on strawberries, which later revealed that the most abundant fungi among the fruit were *Botrytis* and *Cladosporium* [22].

In the Gulf region, studies conducted on fungal contamination levels are few. A study conducted in Bahrain on 17 types of imported spices showed that black pepper and red chili have the highest fungal contamination. The most encountered fungal type was *Aspergillus*, which was isolated from four types of spices followed by *Penicillium*, which was isolated from two types. *Cladosporium*, *Rhizopus*, and *Trichoderma* were also frequently isolated [23]. In Saudi Arabia, 520 samples of date Rutab were tested for microbial spoilage. The two main spoilage-causing agents were *Penicillium* and *Cladosporium* [24]. Despite the various sample types, the results of the two studies are in harmony with our results.

This study illustrates the high level of fungi present in the food products that are consumed daily. However, there are many steps to take that can help avoid human exposure to such microorganisms and conserve imported fruits and vegetables for longer periods of time. Among the required steps are the implementation of programs such as hazard analysis and critical control point (HACCP) in places where food is processed and produced. Programs such as HACCP help to establish the critical control points used to monitor food quality continuously and they help to provide appropriate training to food handlers at all food production stages. Although such measures are costly, they are necessary to ensure appropriate food quality control [25,26].

## Conclusion

Food spoilage monitoring and spoiling agents’ identification is crucial steps in food management programs. Food security is a global concern in both developed and developing countries. The world population has increased from 1.5 to 6.9 billion between 1900 and 2000. This booming comes with an increase in food demand. Fresh fruits and vegetables microbial spoilage causes huge economic losses every year in Qatar and all around the world. Development of spoilage control methods requires first a good knowledge of existing spoiling agents in each country and about the factors that control their growth. It is clear in this analysis that the displaying practices and the prices of the commodities are not food spoilage affecting factors in Qatar, rather fresh produces country of origin and locally produced crops require further attention.

## Authors’ Contributions

IS and RA conceived and designed the experiments and wrote the manuscript. IS performed the experiments. Both authors read and approved the final manuscript.
